# C3 glomerulonephritis and thrombotic microangiopathy of renal allograft after pulmonary infection in a male with concomitant two *complement factor I gene* variations: a case report

**DOI:** 10.1186/s12882-018-0952-z

**Published:** 2018-06-25

**Authors:** Jiqiu Wen, Wei Wang, Feng Xu, Jun Sun, Jinsong Chen, Xuefeng Ni

**Affiliations:** 1 National Clinical Research Center of Kidney Diseases, Jinling Hospital, Nanjing University School of Medicine, East Zhongshan Road 305, Nanjing, 210000 China; 2 National Clinical Research Center of Kidney Diseases, Jinling Hospital, Nanjing Medical University, East Zhongshan Road 305, Nanjing, 210000 China; 3 Running Gene Inc, 35 Northern Garden Street, Haidian District, Beijing, 100089 China

**Keywords:** Kidney allograft, Complement factor I, Mutation

## Abstract

**Background:**

It has been suggested that C3 glomerulonephritis (C3GN) and atypical hemolytic-uremic syndrome (a stereotypical phenotype of thrombotic microangiopathy), two rare entities caused by complement alternative pathway dysregulation share overlapping genetic origin and can be triggered by infections.

**Case presentation:**

We report a case of concomitant C3GN and thrombotic microangiopathy (TMA) after pulmonary infection in a young male receiving kidney transplantation. Genetic assessment revealed two missense variations in compound heterozygous form in *CFI gene (complement factor I).* These two variations are segregated with disease in the core family member of this patient. Plasma CFI levels of the patient and family members were all in normal range. We considered that these two variations only impair CFI function rather than its quantity in the serum.

**Conclusion:**

Our case supports that C3GN and TMA shared overlapping genetic variations and might be triggered by infection in genetically susceptible patients after kidney transplantation.

## Background

It has been increasingly recognized that a subset of patients who were previously diagnosed with membranoproliferative glomerulonephritis (MPGN) and recurred shortly after kidney transplantation (KT) can be recategorized into C3 glomerulopathy. The etiology of which remains elusive, however, some studies have suggested that genetic mutations leading to complement dysregulation play a critical role [1, 2]. Likewise, genetic deficiency in the complement system that results in aberration of the alternative pathway has also been implicated in the pathogenesis of thrombotic microangiopathy (TMA) [3], a typical clinical presentation of which is atypical hemolytic-uremic syndrome (aHUS). For example, it has been reported that *diacylglycerol kinase ε (DGKE) gene* mutation caused a histologic TMA/MPGN overlap pattern [4]. The complement system comprises a host of complement proteins and complement regulators, including complement factor I (CFI), factor H, decay-accelerating factor, membrane cofactor protein and others. Of note, CFI encoded by *CFI gene* is a serine proteinase in the complement pathway that is responsible for cleaving and inactivating the activities of C4b and C3b [5]. The delicate regulation between complement activation and inactivation mediated by complement regulators maintains homeostasis of the complement system. Any genetic or acquired factors that affect the complement regulators may cause complement over-activation and related damages.

Herein, we report a rare case of a young male with a diagnosis of concomitant C3GN and TMA in the renal allograft that harbored two variants of compound heterozygous form in *CFI gene*, while his father and mother who are carriers of only one variant were asymptomatic.

## Case presentation

A 32-year-old male was referred to our hospital for elevated level of serum creatinine (Scr) (3.71 mg/dl) and proteinuria (3+) following a previous deceased cardiac donor-derived KT due to an unidentified cause of end-stage renal disease (ESRD). Laboratory workups and results of diagnostic procedures performed are summarized in Table 1. He underwent a successful KT 26 months ago with Scr at discharge 0.9 mg/dl with an immunosuppressive protocol consisting of prednisone, mycophenolate mofetil and tacrolimus. Renal allograft function remained stable and urine analyses were always normal from discharge to 24 months after operation. Two months prior to this admission, he was hospitalized for fever and cough at another hospital. He was diagnosed with mild pulmonary infection and treated with azithromycin and ceftazidime. His pulmonary symptoms abated after a week antibiotic treatment while his serum Scr increased and proteinuria (3+) occurred. Furthermore, his blood platelet count also decreased to 34 × 10^9^/L. Forty days prior to this admission, a renal allograft biopsy was performed. He was managed with intravenous antibiotics and immunosuppression enhancement by increasing the dosage of mycophenolate mofetil. His Scr level decreased initially with a nadir of 1.58 mg/dl, but elevated progressively with increased proteinuria (11.38 g/24 h). He denied family history of any kidney diseases or inheritable illnesses. A repeat kidney biopsy was performed in our hospital. Written informed consent to publish this case was obtained from this patient.

**Table 1:** Laboratory workups and results of diagnostic procedures

Tested parameters (Patient)	Result (range)
Urine protein (g/24 h)	4.16 (< 0.4)
Hemoglobin (g/L)	101 (130–175)
Platelet count	74 × 10^9 (100–300 × 10^9^)
Serum C3 (g/L)	0.76 (0.8–1.8)
Serum C4 (g/L)	0.1 (0.1–0.4)
Serum CFH (ug/ml)	360.7 (200–1000)
Serum CFH antibody	Negative
Serum C3 nephrotic factor	Negative
DSA	Negative
ADAMTS13 activity (ng/ml)	827.1 (424–1098)
ADAMTS13 antibody (Au/ml)	7.68 (< 27)
Vascular cell adhesion molecule (ng/ml)	1192.73 (300–1000)
Anti-cardiolipin antibody	Negative
Cold agglutinin (mg/L)	20 (< 192)
HBV/HCV/HIV	Negative
Serum immunofixation electrophoresis	Negative
Bone marrow aspirate	Negative
Serum CFI quantity (ug/ml)	Normal range: 16–75
The patient	53.84
Patient’s father	20.2
Patient’s mother	36.6
Patient’s sister	56.6

Abbreviations: ADAMTS13, a disintegrin and metalloproteinase with a thrombospondin type 1 motif, member 13; CFH, complement factor H; HBV, hepatitis B virus; HCV, hepatitis C virus; HIV, human immunodeficiency virus; DSA, donor-specific antibody

**Fig. 1:**
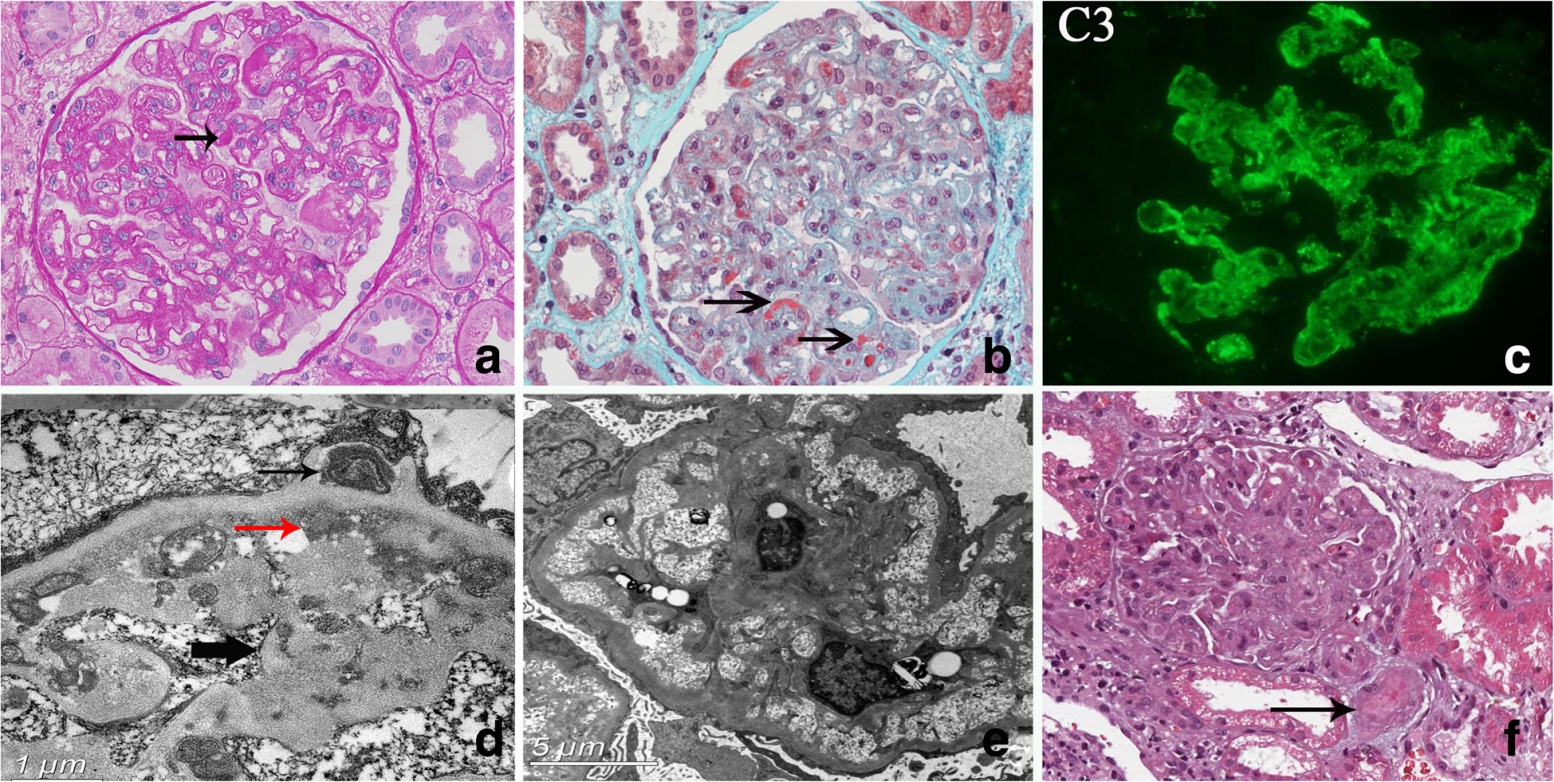
Pathological examination of renal biopsy specimen. **a** PAS revealed an intra-capillary PAS-positive material in a glomerulus (arrow). Extensive capillary duplication can also be observed (PAS × 400). **b** Extensive fuchsinophilic deposits in the subendothelial (long arrow) and mesangium (short arrow) were seen (Masson× 400). **c** Immunofluorescence staining for C3 is strongly positive in the mesangium and along the basement membrane. **d** Electron microscopy demonstrated extensive subendothelial (red arrows) and subepithelial electron-dense deposits (thin black arrow). New membrane formation (fat black arrow) and wide spread foot process effacement are evident. **e** Subendothelial lucency, a characteristic finding in TMA, can be seen in the glomerular tuft. **f** Review of the first biopsy slide provided. The staining quality is suboptimal, however, thrombus (arrow) at arteriole can be clearly identified. The glomerulus exhibited MPGN-like pattern (Masson× 400)

Light microscopy showed multiple periodic acid-Sciff stain (PAS)-positive materials in the capillary lumens (Fig. 1a). Capillary wall duplication was obvious and diffuse (Fig. 1a). Masson trichrome stain revealed extensive fuchsinophilic deposits in the subepithelial, subendothelial and mesangial spaces (Fig. 1b). There were no peritubular capillaritis, endotheliatis, tubulitis nor glomerulitis, excluding the possibility of antibody and T-cell-mediated rejection. Histological signs of calcineurin-inhibitor toxicity, such as band-like fibrosis, isometric vacuolization of the tubules and hyaline deposits in the arterioles were not present.

Immunofluorescence study indicated prominent C3 positivity (3+) along the basement membrane and in the mesangium (Fig. 1c) in all the 6 glomeruli examined, while IgA, IgG, IgM and C1q and C4d staining were all negative.

Electron microscopy examination of 2 glomeruli showed widespread foot process effacement and electron-dense deposits in the subendothelial and subepithelial spaces (Fig. 1d). Furthermore, mesangial proliferation which protruded into capillary basement membrane caused the double contours observed in PAS staining. Subendothelial lucency, which is characteristic of TMA, was also present (Fig. 1e). No signs of chronic antibody-mediated rejection (ie. peritubular capillary multilayering), Based on these findings, a diagnosis of C3GN combined with TMA was rendered.

Retrospective review of the 1st biopsy slides (X.F.) indicated similar light microscopy findings (Fig. 1f). Immunofluorescence showed only prominent C3 staining with negative staining for other immunoglobulins and C4d. No electron microscopy study of the first allograft biopsy was performed.

**Fig. 2:**
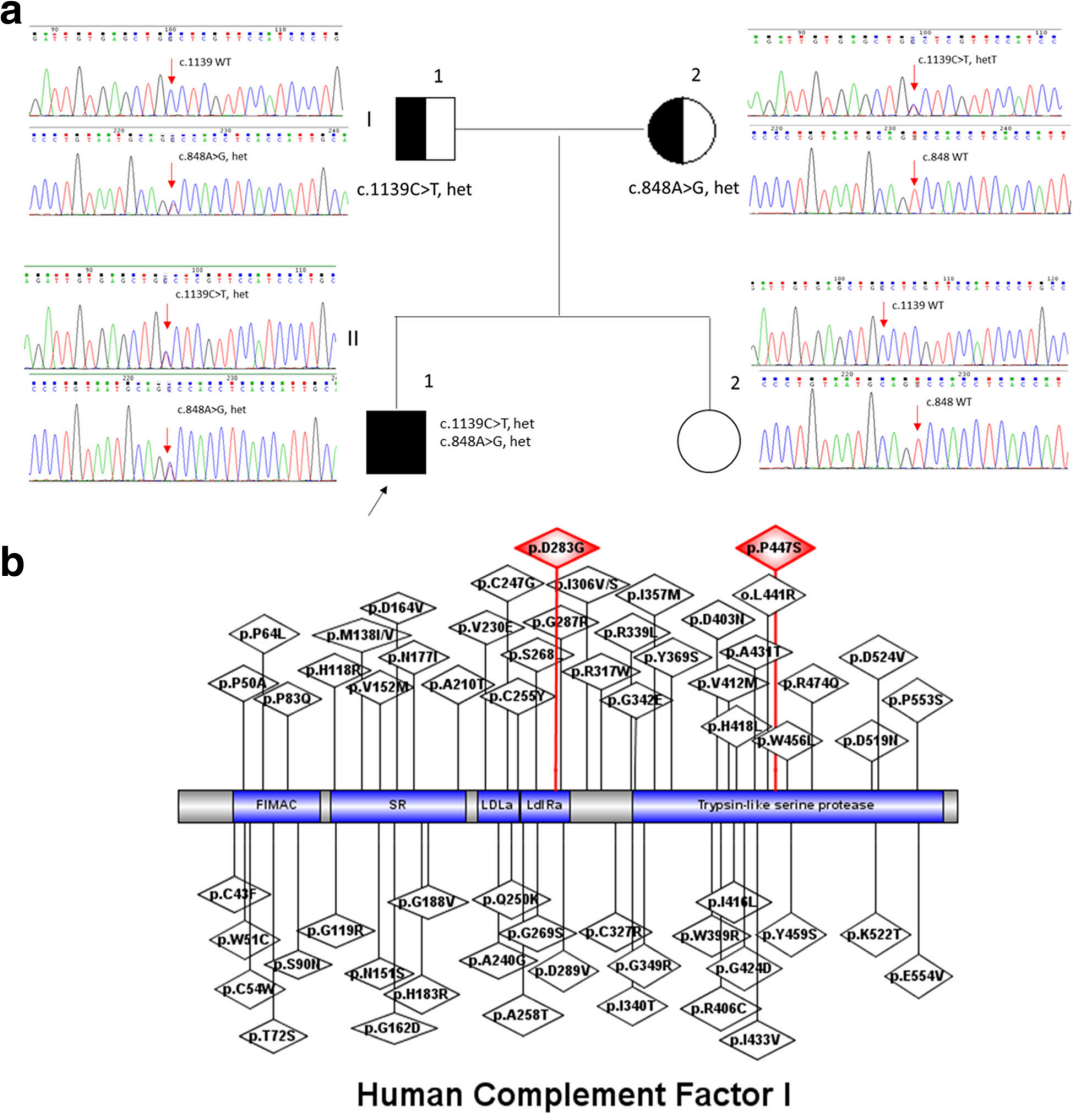
**a** Pedigree and chromatograms of *CFI gene* sequence alterations.Our patient’s father and mother are heterozygous for the c.848A > G and c.1139G > T variants respectively while his sister has neither of the two variants. **b** Schematic representation of main protein domains of CFI protein. FIMAC is the factor I membrane attack complex. SR is the scavenger receptor cysteine-rich domain. Ldla represents low density lipoprotein receptor class A domain. Ldlra is low-density lipoprotein receptor domain class A

Genetic testing for the major genes in complement pathway related with renal disease (C3, CFB, CFH, CFHR1, CFHR3, CFHR4, CFHR5, CFI, DGKE) [1, 2] were performed*.* We found two rare missense variants in compound heterozygous form, c.848A > G (p.Asp283Gly) and c.1339C > T (p.Pro447Ser) in the *CFI gene* (NM_000204.3) in the patient while his father and mother were found to harbor only the c.848A > G and c.1339C > T respectively (Fig. 2a). Both parents were phenotypically normal. This patient’s unaffected sister had neither of the 2 variants. No variants were identified in the other complement cascade protein genes commonly screened. Nevertheless, quantitative measurement of plasma CFI of the patient and his unaffected family members showed that their plasma CFI levels were all in normal range (Table 1).

Our patient was treated with 2 sessions of plasma exchange, but no clinical improvement was achieved as indicated by persistent nephrotic-range proteinuria and progressive elevation of Scr. After approximately one-year follow-up, this patient was in dialysis.

## Discussion and conclusions

There are 2 unique aspects of this particular case. First, C3GN and TMA, both rare diseases, were present simultaneously in this patient. Moreover, the possibility that TMA in this patient was caused by allograft rejection or calcineurin-inhibitor toxicity could be excluded histologically and clinically, making genetic *CFI* variation the most likely underlying etiology. Second, the compound heterozygosity of the two rare *CFI gene* variants may related with diseases in the patient while the patient’s father and mother, who harbors only one of the *CFI gene* variant, were phenotypically normal, indicating that the disease is autosomal-recessive (Online Mendelian Inheritance in Man, OMIM:610984). Serum CFI concentrations of all family members were normal, indicating that CFI functional impairment rather than quantitative deficiency of CFI protein was probably the underlying cause.

It has now been increasingly appreciated that mutations in genes coding for complement regulators are largely responsible for inheritable or acquired C3G and TMA [1, 2, 6]. In addition, there are reports observing that both diseases could share same mutations. Servais et al. [1] identified 8 patients with complement regulator gene mutations/variants that had been previously reported in patients with aHUS in a cohort of 24 patients with C3 glomerulopathy. In fact, among the 6 patients with *CFI* gene mutations in this series, 5 *CFI gene* mutations have been previously reported in aHUS patients. Some investigators even have proposed that aHUS and C3 glomerulopathy should be lumped together as specific disease entities of the alternative complement pathway [7]. In another investigation, Bu et al [2] observed certain degree of overlapping of genetic mutations in C3 glomerulopathy and TMA, although preponderant cases possessed disparate gene variants. A recent paper reporting expert conclusions on aHUS and C3 glomerulopathy suggested that serum complement and complement regulators should be measured in all aHUS and C3 glomerulopathy patients and genetic testing encompassing the above-mentioned complement regulators should be screened on an individual basis [8].

Genetic analyses of our patient and his family members were also informative. Both of his parents were phenotypically normal with a heterozygous *CFI gene* missense variant. The c.848A > G (p.D283G) in his father results in substitution of the highly conserved Asp residue by Gly at the position 283 in low-density lipoprotein receptor domain class A of the CFI protein, which is identified as an important calcium-binding site in this domain (Fig. 2b). *CFI gene* variation in this highly-conserved domain has been demonstrated to impair CFI function as indicated by normal serum CFI quantity [9, 10]. The other missense variant, c.1339C > T in his mother results in substitution of the Pro residue by Ser at position 447, which is located in the trypsin-like serine protease domain of CFI (Fig. 2b). Several deleterious missense variations have been reported in these two domains recorded in HGMD database (http://www.hgmd.cf.ac.uk/). As far as we are aware, these 2 variants of *CFI gene* have not been reported in patients with C3 glomerulopathy or TMA.

It is being increasingly recognized that a host of patients with complement factor gene variations only develop diseases or experience exacerbation after various triggers, one important factor of which is infections [11]. A classic example of this paradigm is aHUS, which typically occurs in patients with genetic complement factor variation after various infections [11]. It is highly likely that our genetically susceptible patient develop C3GN and TMA after pulmonary infection.

Our patient failed to respond to plasma exchange therapy which is currently a main-stay treatment modality in areas without access to eculizumab, a humanized monoclonal antibody against terminal portion of the complement cascade [12]. Sellier-Leclerc *et al* [13] reported that half of the patients with *CFI gene* mutation rapidly evolved to ESRD and plasma therapy had only modest efficacy in aHUS patients with complement regulator gene mutations. In fact, the efficacy of plasma exchange for C3 glomerulopathy is dubious as considered by most experts [8]. Although eculizumab has been demonstrated to be effective in the treatment of aHUS [14] and C3 glomerulopathy [15], this drug is not universally accessible.

In conclusion, we report an interesting case of C3GN and TMA of a transplanted kidney after pulmonary infection in a young male with two variations in the *CFI gene* that presumably resulted in CFI functional deficiency. Our case supports that C3GN and TMA shared overlapping genetic variations and might be triggered by infection in genetically susceptible patients.

## Abbreviations

aHUS: Atypical hemolytic-uremic syndrome
C3GN: C3 glomerulonephritis
CFI: Complement factor I
ESRD: End-stage renal disease
KT: Kidney transplantation
MPGN: Membranoproliferative glomerulonephritis
OMIM: Online Mendelian Inheritance in Man
PAS: Periodic acid-Sciffstain
Scr: Serum creatinine
TMA: Thrombotic microangiopathy
